# The neutral amino acid transporter SLC7A10 in adipose tissue, obesity and insulin resistance

**DOI:** 10.3389/fcell.2022.974338

**Published:** 2022-09-12

**Authors:** Regine Åsen Jersin, Laura Roxana Jonassen, Simon Nitter Dankel

**Affiliations:** ^1^ Hormone Laboratory, Haukeland University Hospital, Bergen, Norway; ^2^ Mohn Nutrition Research Laboratory, Department of Clinical Science, University of Bergen, Bergen, Norway

**Keywords:** solute carriers, adipose tissue, obesity, amino acids, insulin resistance, metabolism, adipocyte subtypes

## Abstract

Obesity, insulin resistance and type 2 diabetes represent major global health challenges, and a better mechanistic understanding of the altered metabolism in these conditions may give improved treatment strategies. SLC7A10, a member of the SLC7 subfamily of solute carriers, also named ASC-1 (alanine, serine, cysteine transporter-1), has recently been implicated as an important modulator of core processes in energy- and lipid metabolism, through its particularly high expression in adipocytes. In human cohorts, adipose *SLC7A10* mRNA shows strong inverse correlations with insulin resistance, adipocyte size and components of the metabolic syndrome, strong heritability, and an association with type 2 diabetes risk alleles. SLC7A10 has been proposed as a marker of white as opposed to thermogenic beige and brown adipocytes, supported by increased formation of thermogenic beige adipocytes upon loss of *Slc7a10* in mouse white preadipocytes. Overexpression of SLC7A10 in mature white adipocytes was found to lower the generation of reactive oxygen species (ROS) and stimulate mitochondrial respiratory capacity, while SLC7A10 inhibition had the opposite effect, indicating that SLC7A10 supports a beneficial increase in mitochondrial activity in white adipocytes. Consistent with these beneficial effects, inhibition of SLC7A10 was in mouse and human white adipocyte cultures found to increase lipid accumulation, likely explained by lowered serine uptake and glutathione production. Additionally, zebrafish with partial global Slc7a10b loss-of-function were found to have greater diet-induced body weight and larger visceral adipocytes compared to controls. However, challenging that SLC7A10 exerts metabolic benefits only in white adipocytes, suppression of SLC7A10 has been reported to decrease mitochondrial respiration and expression of thermogenic genes also in some beige and brown adipocyte cultures. Taken together, the data point to an important but complex role of SLC7A10 in metabolic regulation across different adipose tissue depots and adipocyte subtypes. Further research into SLC7A10 functions in specific adipocyte subtypes may lead to new precision therapeutics for mitigating the risk of insulin resistance and type 2 diabetes.

## Introduction

**FIGURE 1:**
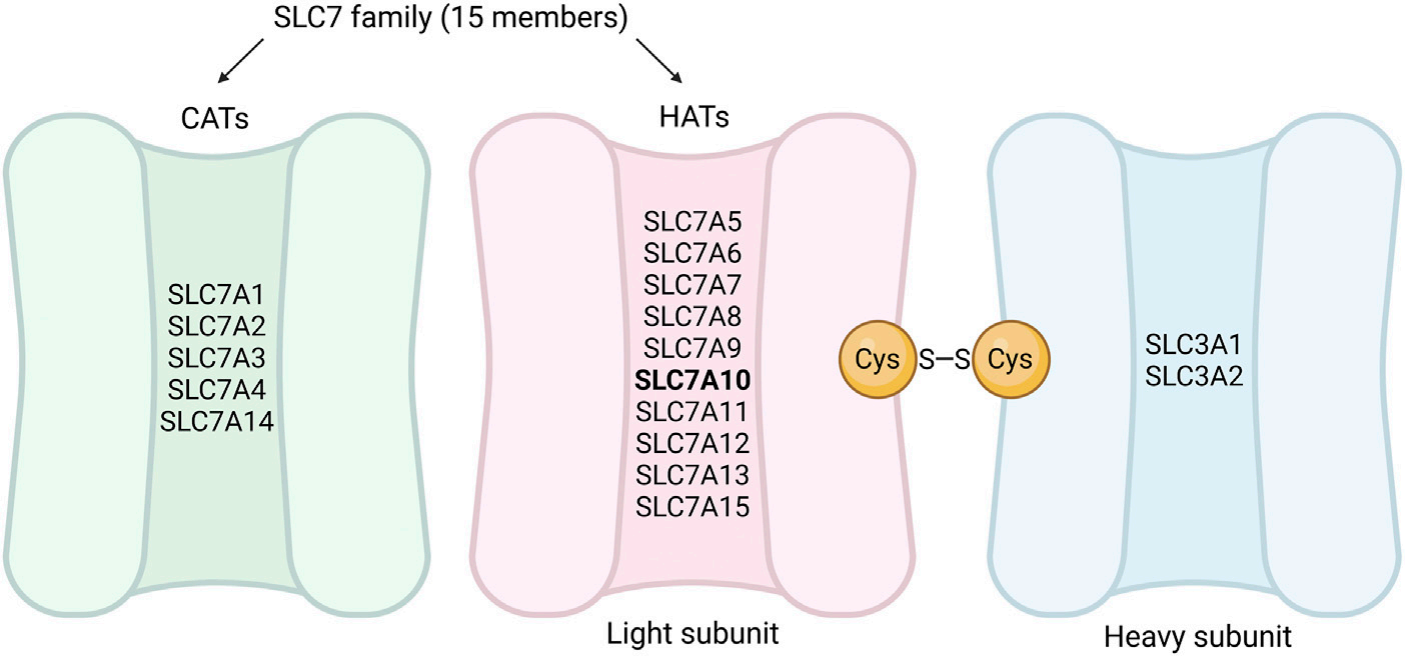
Subclassification of the SLC7 family into CATs and HATs. The SLC7 family can be divided into two subgroups, CATs (represented in green) and HATs (represented in pink). SLC7A1-4 and SLC7A14 are within the CATs subgroup, and SLC7A5-SLC7A13 and SLC7A15 are the light subunits in the HATs subgroup (SLC7A10 highlighted with bold text). The HATs generally associate with a heavy subunit from the SLC3 family (represented in blue) through a conserved disulfide bond (cysteine residues represented as yellow circles). The figure is simplified and not representative of the sizes of the CATs and HATs, nor the positioning of the disulfide bond. CATs, cationic amino acid transporters; HATs, hetero (di) meric amino acid transporters; Cys, cysteine. Figure created in BioRender.com.

Amino acids are involved in numerous cellular processes including synthesis of biomolecules as well as energy- and lipid metabolism (Wu, 2010; Saha et al., 2014). Cellular utilization of amino acids partly depends on their uptake from the circulation, which occurs via different transmembrane amino acid transporters with affinity for specific amino acids (Bröer and Gauthier-Coles, 2022). The biological importance of amino acid transporters is reflected in various diseases associated with their altered function (Bröer and Palacín, 2011; Kandasamy et al., 2018; Errasti-Murugarren and Palacín, 2021; Cibrian et al., 2022). Amino acid transporters are part of the solute carrier (SLC) superfamily. Depending on how rigorously the different genes are validated on the RNA and/or protein level, the SLC superfamily consists of more than 450 transport proteins, and these are in turn divided into subfamilies based on sequence similarity (Perland and Fredriksson, 2017; Schaller and Lauschke, 2019; Pizzagalli et al., 2021; Saier et al., 2021). The SLC7 subfamily consists of 15 members (SLC7A1-SLC7A15) (Fotiadis et al., 2013) and is divided into two subgroups: cationic amino acid transporters (CATs) and hetero (di)meric amino acid transporters (HATs) (Fairweather et al., 2021; Nicolàs-Aragó et al., 2021) (Figure 1). SLC7A1-4 and SLC7A14 are within the CAT subgroup, which comprises amino acid transporters that do not require binding to an additional protein to function (Fotiadis et al., 2013) (Figure 1). SLC7A5-SLC7A13 and SLC7A15 are classified within the HAT subgroup, as they are thought to require a binding partner to localize to the plasma membrane or for transport activity (Fotiadis et al., 2013) (Figure 1). In general, the HATs may associate with a heavy subunit (either SLC family 3 member 1 (SLC3A1) or member 2 (SLC3A2)) through a conserved disulfide bond (Fairweather et al., 2021) (Figure 1).

Members of the SLC7 family have been implicated in various diseases (Feliubadaló et al., 1999; Torrents et al., 1999; Kaira et al., 2008; Sakata et al., 2009; Wang et al., 2011, 2013; Tărlungeanu et al., 2016; Knöpfel et al., 2019; Cascio et al., 2020), including metabolic diseases (Small et al., 2011; Jersin et al., 2021). In the present review we focus on the HAT SLC7A10, which has a particularly high expression in adipocytes and emerged as an important player in metabolic diseases related to obesity, insulin resistance and type 2 diabetes (Small et al., 2011; Jersin et al., 2021). Following a brief summary of key roles of adipose tissue in metabolic homeostasis, we discuss the current knowledge of how SLC7A10 modulates adipocyte function (Arianti et al., 2021; Jersin et al., 2021; Suwandhi et al., 2021) and propose avenues for further research.

## Roles of adipose tissue in metabolic homeostasis

Metabolic diseases including obesity, type 2 diabetes and cardiovascular disease are strongly associated with reduced insulin receptor function on target cells, often concomitant with elevated blood insulin levels, referred to as a state of insulin resistance (Lee and Olefsky, 2021). Insulin resistance has profound physiological consequences, since insulin largely controls the metabolism of amino acids, glucose and fatty acids in key metabolic organs including liver, muscle, adipose tissue and brain (Petersen and Shulman, 2018). Clinically, insulin resistance typically manifests in increased blood glucose levels (hyperglycemia), as well as elevated blood pressure and blood triacylglycerol levels, and reduced blood high-density lipoprotein cholesterol (HDL-C) levels (Petersen and Shulman, 2018). Cellular mechanisms that enhance whole-body insulin sensitivity may therefore help prevent the development of metabolic diseases. The gold standard for measuring insulin sensitivity is the hyperinsulinemic euglycemic clamp, which determines the glucose infusion rate (GIR) required to maintain normal blood sugar during a constant insulin infusion (where a higher GIR indicates higher insulin sensitivity) (DeFronzo et al., 1979).

Adipose tissue has emerged as a key determinant of whole-body insulin sensitivity and energy homeostasis (Sakers et al., 2022). The main adipose tissue depots are found subcutaneously and viscerally, comprising the “white” adipose tissue, while smaller amounts of the highly thermogenic “brown” adipose tissue is found in the neck and between shoulder blades (Sakers et al., 2022). Moreover, “beige” adipocytes can form within white adipose tissue upon adrenergic and other stimuli to increase thermogenesis and regulate energy homeostasis, such as in response to cold exposure (Chen et al., 2019). Thermogenesis is a process in which chemical energy is dissipated as heat via mitochondrial uncoupling, which in beige and brown adipocytes may largely occur via uncoupling protein 1 (UCP1) (Heaton et al., 1978; Aquila et al., 1985). Additionally, functionally distinct adipocyte subtypes within the main categories of white, beige and brown adipocytes have been described, including white and beige adipocytes with a comparatively higher glycolytic activity than other subtypes (Chen et al., 2019; Lee et al., 2019). At least some of these adipocyte subtypes are present in different proportions in subcutaneous and visceral adipose tissue (Lee et al., 2019; Raajendiran et al., 2019). In any case, both the subcutaneous and visceral white adipose tissue of individuals with insulin resistance typically show enlarged adipocytes (hypertrophy) as well as increased numbers of pro-inflammatory macrophages, in turn associated with systemic low-grade inflammation (Klöting et al., 2010).

These physiological changes, and the consequent risk of type 2 diabetes, are strongly associated with reduced blood levels of the adipocyte-derived peptide hormone adiponectin (Arita et al., 1999; Li et al., 2009; Klöting et al., 2010; Matsuzawa, 2010). Adiponectin, a 30 kilodalton peptide, which forms large homodimers and structurally resembles complement factor C1q, is selectively produced by adipocytes in white adipose tissue (Scherer et al., 1995) and capable of reversing the insulin resistance associated with both obesity and lipodystrophy (Berg et al., 2001; Yamauchi et al., 2001). Strikingly, transgenic obesity- and diabetes-prone mice made to overexpress adiponectin in adipose tissue were found to develop extreme obesity but to retain a low size of individual adipocytes and remain insulin sensitive (Kim et al., 2007). Thus, adiponectin and other insulin-sensitizing factors may promote a metabolically healthy fat storage in subcutaneous as well as visceral adipose tissue, by stimulating the generation of new adipocytes (hyperplasia) and thereby preventing excessive expansion of individual adipocytes (hypertrophy), referred to as the adipose tissue expandability hypothesis (Vishvanath and Gupta, 2019). Mechanistically, adiponectin has been reported to stimulate fatty acid oxidation in skeletal muscle cells, suppresses glucose production in hepatocytes, and has anti-inflammatory and anti-apoptotic effects (Yokota et al., 2000; Berg et al., 2001; Yamauchi et al., 2002; Li et al., 2009; Ohashi et al., 2010; Miller et al., 2011).

## Emerging metabolic functions of SLC7A10

SLC7A10 is a sodium-independent small neutral amino acid transporter, also named alanine, serine, cysteine transporter-1 (ASC-1) (Fukasawa et al., 2000). This carrier was primarily investigated due to its function as a transporter with high affinity for both glycine and serine in the brain (Fukasawa et al., 2000; Nakauchi et al., 2000), where these amino acids are important for N-methyl-D-aspartate (NMDA) receptor regulation (Rutter et al., 2007; Rosenberg et al., 2013). The NMDA receptor has previously been linked to diseases such as schizophrenia and Alzheimer’s (Cheng et al., 2021), and several studies have therefore investigated the effects of pharmacologic SLC7A10 inhibition on SLC7A10 function related to the transport of both D- and L-stereoisomers of small neutral amino acids in the brain (Brown et al., 2014; Sason et al., 2016; Errasti-Murugarren and Palacín, 2021). SLC7A10 is present in both astrocytes and neurons (Mikou et al., 2020) and has been found to be responsible in particular for D-serine and glycine release, as well as D- and L-serine uptake (Rutter et al., 2007; Rosenberg et al., 2013; Safory et al., 2015; Sakimura et al., 2016; Sason et al., 2016; Mikou et al., 2020). Reduced glycine but not D-serine levels were observed in the nervous system of Slc7a10 knockout mice (Safory et al., 2015). Another study using neocortical slices from Slc7a10 knockout mice found lower D-serine release compared to wildtype counterparts (Sason et al., 2016). However, that study measured D-serine release after preloading the brain slices with radiolabeled D-serine (Sason et al., 2016), while other studies added radiolabeled D-serine extracellularly and used the human embryonic kidney cell line HEK293 (Rutter et al., 2007; Rosenberg et al., 2013; Brown et al., 2014). These differences in experimental methods and models may, at least in part, explain the reported differences in the preferred amino acids carried by SLC7A10 as well as the direction of transport.

SLC7A10 mostly received attention for its role in the brain, until Ussar and coworkers in 2014 proposed SLC7A10 as a white adipocyte marker, and reported a 5 times higher SLC7A10 expression in adipose tissue compared to the brain (Ussar et al., 2014). An earlier study had also found inverse correlations between *SLC7A10* mRNA expression in subcutaneous adipose tissue and risk factors for metabolic disease (e.g., body-mass index (BMI), fasting glucose, insulin and triacylglycerols) (Small et al., 2011). This latter analysis was motivated by reduced subcutaneous adipose *SLC7A10* expression in carriers of risk alleles in the type 2 diabetes-associated locus near the *KLF14* gene (Small et al., 2011). Notably, the authors also observed a positive correlation between the adipose *SLC7A10* expression and circulating levels of adiponectin (Small et al., 2011). Consistently, in their systematic search for white adipocyte markers, Ussar and colleagues initially identified SLC7A10 by assessing genes with the strongest correlation with adiponectin gene expression across the entire transcriptome in white adipose tissue (Ussar et al., 2014).

More recently, in an independent systematic transcriptome screen for genes with altered adipose mRNA expression in obesity, *SLC7A10* was identified as a top hit with different expression between subcutaneous and visceral adipose tissue as well as increased expression in subcutaneous adipose tissue 1 year after profound fat loss due to bariatric surgery (Jersin et al., 2021). Among the top hits identified from this systematic screen, *SLC7A10* also showed the highest mRNA expression in mature adipocytes isolated from human adipose tissue (Jersin et al., 2021). Decreased subcutaneous as well as visceral adipose expression of *SLC7A10* mRNA in insulin resistance was confirmed by directly comparing BMI-matched people with obesity who were either insulin resistant or insulin sensitive determined by hyperinsulinemic euglycemic clamp (Jersin et al., 2021). Taken together, these correlative data point to *SLC7A10* as an important modulator of insulin resistance and metabolic health via white adipocytes. To evaluate a possible causal role of altered *SLC7A10* expression in obesity, an Slc7a10b Zebrafish loss-of-function model was subjected to overfeeding for 2 months (Jersin et al., 2021). Because two isoforms of Slc7a10 exist in zebrafish (Slc7a10a as well as Slc7a10b), this model should be considered a partial global knockout of Slc7a10. A partial knockout was performed due to the severe phenotype (tremors, seizures and post-natal death) observed in mice with a complete global knockout of Slc7a10 (Xie et al., 2005). Moreover, Slc7a10b was chosen over Slc7a10a due to its slightly higher sequence identity (76% over 74%) with the human *SLC7A10* (found using the T-coffee multiple sequence alignment tool (Notredame et al., 2000)). Compared to wildtypes, the fish with impaired Slc7a10b function gained 38% more body weight and had on average 49% larger visceral adipocytes (Jersin et al., 2021), suggesting that Slc7a10 confers protection against excess fat storage and adipocyte hypertrophy.

## Role of SLC7A10 in adipocyte energy metabolism

**FIGURE 2:**
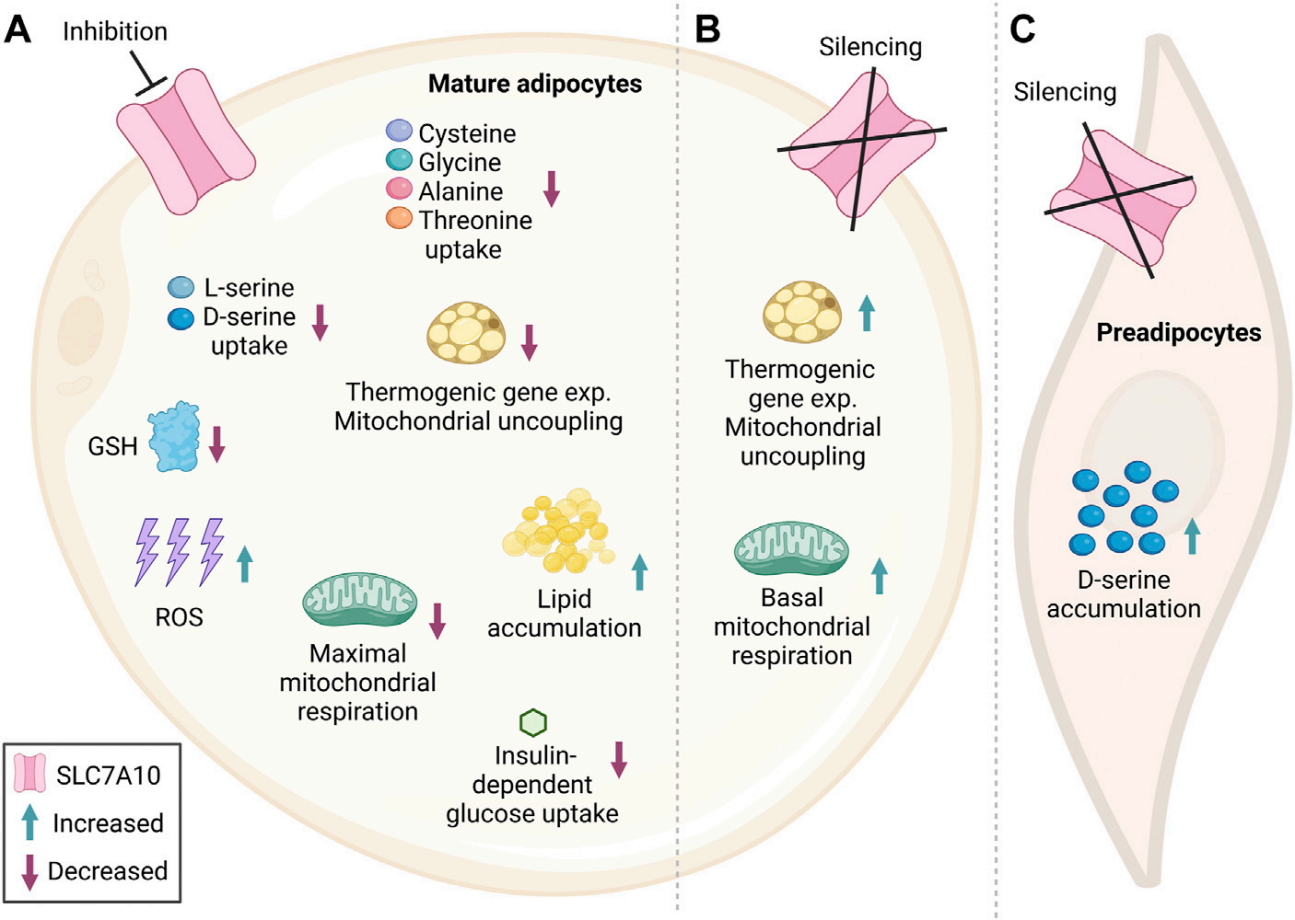
Simplified summary figure of the metabolic consequences of impaired SLC7A10 function in adipocytes. Several studies have implicated SLC7A10 as an important player in adipocyte metabolism. Blunted SLC7A10 in cultured human subcutaneous and mouse fat cells reduces serine uptake and the total level of the antioxidant glutathione, which may further have caused the observed increase in ROS generation **(A)**. Moreover, inhibition of the carrier has been found to reduce consumption of cysteine, glycine, alanine and threonine in adipocytes isolated from human deep neck adipose tissue **(A)**. Impairment of SLC7A10 also counteracted insulin-stimulated glucose uptake in cultured human subcutaneous and murine adipocytes **(A)**. Furthermore, the high ROS levels might, at least in part, promote the observed increase in lipid accumulation and associated reduction in mitochondrial respiration seen in cultured human subcutaneous and mouse adipocytes, although the sequence of these events need to be established in further studies **(A)**. Inhibition of Slc7a10 function has also been shown to decrease the expression of thermogenic marker genes and mitochondrial uncoupling in differentiated human SGBS cells and adipocytes from deep neck stimulated with cAMP **(A)**. In differentiated mouse adipocytes where Slc7a10 was silenced already at the preadipocyte stage, thermogenic gene expression, mitochondrial uncoupling and basal mitochondrial respiration was increased compared to control **(B)**. Undifferentiated mouse preadipocytes with partial Slc7a10 knockout showed increased D-serine accumulation **(C)** highlighting a complex role for SLC7A10 in the regulation of (pre) adipocyte metabolism which requires further investigation. Subcellular localization is not accounted for. GSH, Glutathione; ROS, reactive oxygen species; exp, expression. Figure created in BioRender.com.

Recent studies have explored the metabolic functions of SLC7A10 in adipocytes by inhibition or overexpression experiments in cell cultures. We here present a summary of how this amino acid carrier mechanistically modulates adipose metabolism and thereby may regulate global insulin responses (Figure 2). To determine which amino acid(s) showed altered uptake when impairing SLC7A10 directly in cultured adipocytes, Jersin and colleagues performed inhibition of the carrier during adipocyte maturation (Jersin et al., 2021). The highly selective inhibitor BMS-466442 (Brown et al., 2014; Torrecillas et al., 2019) caused a potent reduction in D-serine as well as L-serine uptake (Jersin et al., 2021) (Figure 2A). Serine has a central role in one-carbon metabolism, where it serves as a donor of one-carbon groups to the folate cycle (Davis et al., 2004), which is essential for regeneration of the cofactors NADPH and NADH as well as ATP (Tedeschi et al., 2013). Additionally, serine is involved in production of the body’s main antioxidant glutathione (GSH) through the transsulfuration pathway as part of the folate cycle (Ducker and Rabinowitz, 2017; Kurniawan et al., 2020). GSH is important for scavenging reactive oxygen species (ROS), which are formed in cells during oxidative stress (Gutscher et al., 2008; Murphy, 2012). When mitigating oxidative stress, GSH is oxidized to GSSG, and NADPH is important for regenerating GSH by reducing GSSG (Newman and Maddocks, 2017). Maintenance of basal ROS levels is thought to be an important signaling component for normal adipocyte differentiation, while too high levels are considered a trait of adipocyte dysfunction (Ghaben and Scherer, 2019). In line with serine’s role as a precursor for GSH (Mattaini et al., 2016; Kurniawan et al., 2020), SLC7A10 inhibition in adipocytes lowered the intracellular levels of GSH, concomitant with increased ROS generation (Jersin et al., 2021) (Figure 2A). Conversely, overexpression of SLC7A10 led to higher total GSH levels and reduced ROS production, indicating that SLC7A10 mitigates oxidative stress in adipocytes (Jersin et al., 2021). Of note, a previous metabolomics analysis of Slc7a10 knockout mice revealed no significant differences in the level of brain metabolites involved in the glutathione pathway compared to wildtype controls (Safory et al., 2015).

Supporting that SLC7A10 is important for serine transport in adipocytes, another study in mice found that loss of Slc7a10, not in mature adipocytes (Figure 2B), but in proliferating preadipocytes increased intracellular D-serine accumulation (Suwandhi et al., 2021), suggesting that Slc7a10 may also be important for *export* of D-serine (at least when preadipocytes are programmed towards a beige phenotype) (Figure 2C). The bidirectional transport of amino acids is possible since SLC7A10 functions as an antiporter, where it transports one small neutral amino acid in one direction coupled to an amino acid in the opposite direction (Fukasawa et al., 2000). Additionally, SLC7A10 has a facilitative diffusion mode, where it transports small neutral amino acids along their concentration gradient (Fukasawa et al., 2000; Pineda et al., 2004). Hence, SLC7A10 might transport neutral amino acids in or out of adipocytes dependent on the cellular and physiological condition, including feeding or fasting signals. In addition to the SLC7A10-inhibitor effects on serine flux, Arianti and colleagues observed reduced uptake of the other neutral amino acids cysteine, glycine, alanine and threonine by SLC7A10 inhibition in human white adipocytes isolated from deep neck adipose tissue (Arianti et al., 2021) (Figure 2A). However, Jersin and colleagues did not observe any effect on the consumption of these amino acids with Slc7a10 inhibition in cultured and differentiated abdominal white subcutaneous adipocytes (Jersin et al., 2021). This discrepancy might be due to inherent functional differences between adipocyte cultures from the neck and abdomen. Arianti *et al.* used adipose samples from human deep neck, likely representing functionally distinct adipocyte subtypes compared to the human subcutaneous abdominal adipocytes used by Jersin and colleagues (e.g., a greater thermogenic capacity in the former) (Tóth et al., 2020; Arianti et al., 2021). Differences in experimental approaches may also have affected the concentration gradients of amino acids intra- and extracellularly, and the measured amino acid fluxes. Jersin et al. inhibited SLC7A10 and directly measured intracellular uptake of the extracellularly added radiolabeled D- or L-serine (Jersin et al., 2021). In contrast, in preadipocytes from mice with *Slc7a10* knockdown, Suwandhi and coworkers found increased D-serine levels intracellularly and decreased levels extracellularly, while this was not studied in mature adipocytes (Suwandhi et al., 2021). Thus, different culture mediums, cell states, assays and mode of SLC7A10 impairment (transient protein inhibition or partial permanent gene knockout) may all have contributed to the apparent differences in serine compartmentalization.

Emerging evidence supports that cellular serine status influences metabolic homeostasis via insulin action. Mice given a dietary challenge together with 1% D-serine in their drinking water for 8 weeks showed blunted insulin secretion from pancreatic beta cells and hyperglycemia compared to controls not given extra D-serine (Suwandhi et al., 2018). In SLC7A10-inhibited cultured mouse adipocytes with reduced serine uptake, Jersin and colleagues found a marked reduction in insulin-dependent glucose uptake (Jersin et al., 2021) (Figure 2A). The exact mechanism for how a lowered serine uptake might have mediated this effect, and how the effect might vary according to physiological condition, remains unknown. In addition to reducing glucose uptake, pharmacologic inhibition of SLC7A10 was found to increase lipid accumulation in cultured adipocytes compared to controls (Figure 2A), consistent with the generation of larger lipid-laden adipocytes seen in Slc7a10b loss-of-function zebrafish (Jersin et al., 2021). Mechanistically, reduced SLC7A10 activity may promote lipid accumulation at least in part via the increase observed in ROS generation upon SLC7A10 inhibition (Jersin et al., 2021), as elevated ROS can have lipid-storing effects (Jones IV et al., 2016) (Figure 2A). Accordingly, treatment of adipocytes with the ROS scavenger N-acetylcysteine (NAC, which enhances glutathione synthesis) counteracted the lipid storing effect of SLC7A10 inhibition by 50–70%, supporting that increased ROS at least partly mediated the lipid storage (Jersin et al., 2021). Changes in NADPH may here be involved, given the key role of serine in regulating NADPH regeneration and NADPH’s role in lipid synthesis (Ducker and Rabinowitz, 2017). Supporting this hypothesis is the finding that genes involved in NADPH regeneration were upregulated by SLC7A10 inhibition in primary human adipocyte cultures (Jersin et al., 2021). The lipid-storing effect of high ROS may also occur via impaired mitochondrial respiration in adipocytes (Wang et al., 2010), in line with the reduced maximal mitochondrial respiration upon Slc7a10 inhibition observed in human as well as mouse adipocytes in two independent studies (Arianti et al., 2021; Jersin et al., 2021) (Figure 2A), and with the increased mitochondrial respiration in Slc7a10-overexpressing adipocytes (Jersin et al., 2021). Reduced white adipocyte mitochondrial respiration has been observed in several mouse models of obesity (Schöttl et al., 2015). It should be noted that Arianti and colleagues found that SLC7A10 inhibition reduced maximal mitochondrial respiration (Arianti et al., 2021) only in adipocytes that were induced to express UCP1 via treatment with cyclic AMP (cAMP) (a second messenger mediator of β-adrenergic stimulation and fasting responses) (La Vine et al., 1975) (Figure 2A), suggesting contingency on the metabolic/physiologic condition.

Further highlighting a complex role of SLC7A10 in regulating mitochondrial function, Suwandhi and coworkers reported that knockout of Slc7a10 in white mouse preadipocytes isolated from deep neck induced expression of beiging marker genes, along with increased mitochondrial uncoupling (indicative of thermogenesis), as well as increased basal respiration (Suwandhi et al., 2021) (Figure 2B). These data suggest that Slc7a10 serves to prevent the generation of thermogenesis-competent beige adipocytes, in line with SLC7A10 being a specific marker of white as opposed to beige/brown adipocytes (Ussar et al., 2014). Moreover, this apparent inhibitory effect of Slc7a10 on adipocyte beiging supports that the stimulatory effects of SLC7A10 overexpression observed on mitochondrial respiration in white adipocytes (Jersin et al., 2021) may be independent of the mechanisms of adipocyte beiging/browning. Indeed, the initial work by Ussar and colleagues in this regard pointed to low SLC7A10 expression in adipocytes expressing UCP1, a molecular hallmark of beige/brown adipocytes (Ussar et al., 2014). Garcia *et al.* also found reduced expression of *Slc7a10* mRNA in beige adipocytes obtained from white inguinal adipose tissue of obesity-prone mice, following treatment of white adipocytes with the beiging stimulants rosiglitazone and thyroid hormone triiodothyronine (T3) (Garcia et al., 2016). Neither these white nor beige adipocytes showed altered *Slc7a10* mRNA expression upon β-adrenergic stimulation (with isoproterenol which is expected to activate UCP1) (Garcia et al., 2016). In contrast, Arianti and colleagues observed significantly higher expression of *SLC7A10* mRNA following chemical induction of UCP1 expression with 10-h cAMP stimulation in *vitro*-differentiated human subcutaneous adipocytes (SGBS cell line) and primary brown adipocytes obtained from superficial and deep neck adipose tissue (although no significant differences were observed in SLC7A10 protein expression levels between any of the cultures) (Arianti et al., 2021). Additionally, as opposed to the increased mitochondrial uncoupling observed by Suwandhi and coworkers upon knockout of Slc7a10 in white preadipocytes (Suwandhi et al., 2021), Arianti *et al.* found decreased mitochondrial uncoupling and expression of thermogenic marker genes when SLC7A10 was inhibited in deep neck adipocytes (Arianti et al., 2021) (Figures 2A,B). Thus, there are conflicting reports on SLC7A10 as a selective marker of white as opposed to beige/brown adipocytes, and evidence also for a positive role of SLC7A10 in β-adrenergic- and UCP1-dependent adipocyte beiging/browning and thermogenesis. These data might suggest that SLC7A10 plays a metabolic role in certain subtypes of beige and brown adipocytes.

## Discussion and future perspectives

With its high expression in mature white adipocytes and strong inverse correlations with insulin resistance, adipocyte SLC7A10 has recently emerged as an important amino acid transporter in the pathogenesis of obesity-related metabolic disease (Jersin et al., 2021). Subcutaneous adipose *SLC7A10* mRNA expression was found to be highly heritable (heritability index of 0.79), and in part dependent on risk variants in the type 2 diabetes risk locus by the *KLF14* gene (Small et al., 2011). Thus, SLC7A10 might represent a target for personalized intervention to ameliorate insulin resistance. The evidence reviewed here may additionally provide new leads for exploring the functions of SLC7A10 in neurons, astrocytes and other cell types which also express SLC7A10, with possible implications for neurodegenerative diseases.

Regarding the observed metabolic functions of SLC7A10, it is intriguing that inhibition of a factor that shows increased expression during adipocyte maturation results in a further increase in lipid storage (Jersin et al., 2021). A similar phenomenon is seen for adiponectin, which shows decreased circulating concentrations in obesity despite being highly expressed specifically in adipocytes (Arita et al., 1999). In contrast to adiponectin, however, which was found to cause massive weight gain when overexpressed in mice while improving insulin sensitivity (Kim et al., 2007), the data from SLC7A10 impairment in zebrafish as well as in human subcutaneous adipocyte cultures suggest that SLC7A10 might improve insulin sensitivity rather by limiting lipid accumulation in subcutaneous and visceral adipose tissues (Jersin et al., 2021). These findings should be confirmed in additional *in vivo* model systems, particularly to see if increased adipocyte SLC7A10 expression by genetic manipulation would prevent or limit lipid accumulation upon dietary challenge (including changes in adipocyte size), how such effects would relate to whole-body insulin sensitivity, and by what mechanisms this combination of effects might occur. Based on the current evidence reviewed here, Slc7a10 overexpression can be hypothesized to protect against diet-induced obesity and insulin resistance. These models might also reveal novel aspects of a solute carrier-dependent metabolic crosstalk between white adipose tissue and the liver, skeletal muscle and brown adipose tissue.

Moreover, in future work it will be important to clearly map out the specific adipocyte subtypes that depend on SLC7A10 for their prioritization of mitochondrial respiration over progressive lipid accumulation and adipocyte hypertrophy. Recent flow cytometry and single-cell RNA sequencing experiments have revealed different compositions of functionally distinct adipocyte progenitor cells within different adipose tissue depots throughout the body, with relevance for insulin resistance and type 2 diabetes (Schwalie et al., 2018; Lee et al., 2019; Raajendiran et al., 2019). Importantly, Suwandhi and colleagues found that Slc7a10 marks a specific subset of white preadipocytes (Suwandhi et al., 2021). By corollary, the 2-fold higher expression of SLC7A10 in mature visceral compared to subcutaneous adipocytes in humans (Ussar et al., 2014; Jersin et al., 2021) might reflect different proportions of white (pre)adipocyte subtypes within the respective adipose tissue depots, and/or higher expression of SLC7A10 in a distinct adipocyte subtype within the visceral depot. Given the notable UCP1 expression in human visceral adipose tissue as a whole (Lim et al., 2020) and the generally diminished expression of SLC7A10 in brown adipose tissue (Ussar et al., 2014), SLC7A10 may limit lipid storage in specific visceral adipocyte populations that do not express UCP1. In these UCP1-negative cells, this anti-obesity effect could occur via increased mitochondrial respiratory capacity (Schöttl et al., 2015) and UCP1-independent thermogenesis (Granneman et al., 2003). Different adipocyte subtypes/compositions may also help explain some of the conflicting data on SLC7A10 functions, as many different adipocyte models were used in the studies of SLC7A10 reviewed herein, including established cell lines and primary human and mouse cultures. For example, some of the studied cultures might consist of specific subtypes of beiging-competent white adipocytes or brown adipocytes that induce rather than suppress *SLC7A10* expression upon thermogenic stimulation. This possibility could help explain the lack of difference in *SLC7A10* expression between white and brown adipocyte cultures derived from the neck and the co-expression of *SLC7A10* and *UCP1* mRNA in the study of Arianti and colleagues (Arianti et al., 2021), while SLC7A10 was found on the other hand to be anticorrelated to UCP1 expression by Ussar and colleagues (Ussar et al., 2014). Slc7a10 might, for example, play a role in the beige adipocyte subtype recently discovered to have high glycolytic activity and to mediate metabolic adaptation to cold independent of β-adrenergic stimulation (Chen et al., 2019). Moreover, decreased D-serine uptake was observed in already differentiated primary human and 3T3-L1 adipocytes (Jersin et al., 2021), reflecting the effect of Slc7a10 inhibition without changes in proliferation of specific adipocyte subtypes. In contrast, the increased intracellular D-serine accumulation after Slc7a10 knockdown in white preadipocyte cultures (Suwandhi et al., 2021) might rather reflect the increased proliferation of beige adipocytes that could have higher serine uptake via other serine transporters and/or higher cellular D-serine synthesis. Here it is relevant to consider that preadipocytes alter their consumption of specific amino acids throughout differentiation into mature adipocytes (Green et al., 2016; Halama et al., 2016). Bidirectional transport of serine according to cellular/experimental conditions is possible because SLC7A10 functions as an antiporter and has a facilitative diffusion mode that allows transport of amino acids along their concentration gradient (Fukasawa et al., 2000; Pineda et al., 2004).

Finally, it will be important to identify factors that regulate expression and function of SLC7A10, which might provide therapeutic options for activation either by targeting the protein directly or the gene via regulatory mechanisms that control SLC7A10 expression. While therapeutic options that inhibit SLC7A10 have been explored for the potential treatment of neurological disease (e.g., Schizophrenia) (Errasti-Murugarren and Palacín, 2021), including S-methyl-L-cysteine which activates D-serine efflux (although non-selectively) (Ishiwata et al., 2013), the evidence presented in this review should motivate efforts towards also developing potential activators. The branched-chain amino acid D-isoleucine was found to selectively enhance the antiporter function of SLC7A10 and reduce the uptake of serine and glycine into neurons (Rosenberg et al., 2013; Mesuret et al., 2018), which might provide clues for activating SLC7A10 in adipocytes. Finally, the transcription factor KLF14 is one possible regulator of *SLC7A10* mRNA expression (Small et al., 2011), and identification of such transcriptional activators and the upstream signaling pathways could be another therapeutic avenue to explore.

In conclusion, SLC7A10 is a neutral amino acid transporter with a particularly high expression in white adipocytes, cells that play a central role in the development of obesity, insulin resistance and type 2 diabetes. In different adipocyte models, SLC7A10 has been suggested to exert metabolically beneficial effects on energy- and lipid metabolism, consistent with its decreased adipose expression in obesity, insulin resistance and type 2 diabetes in human cohorts. Future studies should determine the physiological consequences of altered adipocyte SLC7A10 expression *in vivo*, and further explore the metabolic functions of SLC7A10 in specific subtypes of white as well as beige/brown adipocytes. These studies may improve our understanding of insulin resistance development, and provide new avenues for precision therapeutics that target SLC7A10 in a genotype- and adipocyte subtype-dependent manner.
